# Case of Extreme Narcotism from Opium and Treatment

**Published:** 1855-09

**Authors:** E. C. Atkinson

**Affiliations:** Dover, Lee Co., Iowa


THE
### IOWA MEDICAL JOURNAL.

VOL. II. KEOKUK, IOWA, AUG. AND SEPT., 1855. NO. VI.

### ORIGINAL COMMUNICATIONS.

### CASE OF EXTREME NARCOTISM FROM OPIUM, AND TREATMENT,

BY E. C. ATKINSON, M. D., DOVER, LEE CO., IOWA.

On the 22d of August, 1854, I was summoned to visit Caroline B****, living in Charleston township, a sprightly little girl five years of age, of red hair, light complexion, and naturally delicate physical constitution. On the day previous she had been attacked with acute dysentery; her symptoms such as usually occur in such cases. I prescribed for her and left for the day. On my return the next morning at 8 o'clock, I found her somewhat worse, —all the morbid symptoms of the day before were present in an increased degree, especially tenesmus, from the effects of which she was continually writhing. I now ordered a powder composed as follows:

℞ Calomel grs. ii.
Dover Powder grs. iv.
Camphor grs. ii.
Ipicac gr. i.

Also, left one and a half drachms of Tinc. Opii. (full strength), directing eight drops of it to be given *per annum* immediately, in an emulsion of Gum Arabic, and oil of sweet almonds; to be repeated every hour until the patient become easy.

Intending to see her again in a short time, I left no further prescription at that time. On my return at 9 o'clock, I found the mother alarmed at the unusual appearance of her child, and learned that immediately after I left she had committed the error by giving the whole of the Tinc. Opii. by injection at once. It had been retained, and symptoms of narcotism began to make their appearance twenty minutes afterwards, and were now rapidly progressing. She was now lying on her back, her countenance pale and livid, her head thrown back, eye-balls turned upwards, iris contracted and fixed, head and upper part of spine warm, extremities cold and mottled, complete anæsthesia of the skin, and suspension of the special senses. Acrid vapors applied to the nostrils elicited little or no manifestations of sensation. There was some perspiration of the face and neck, breathing slow and difficult. Pulse at the wrist accelerated and weak, caroted pulse strong.

I now ordered a large stimulating injeetion, hoping the tincture might not be wholly absorbed, and immediately resorted to the use of cold water, first in form of a douche, over the head and face, subsequently kept a small stream falling from a distance over the face, head, and spine. This treatment was continued with occasional short intermissions until fifteen minutes past 10 o'clock.—She now became considerably revived, answered when loudly spoken to, breathed better, pupils movable, radial pulse fuller and stronger. We now succeeded in making her swallow a dose of castor oil and a cup of strong coffee. I now cupped her spine, used stimulating frictions, sinapisms, warm foot bath, sprinkled her face with cold water, carried her into the air, and in short, made use of every means at hand except showering as at first, to keep her aroused. Nothwithstanding however, at 11 o'clock, she began to relapse, and at 12 her appearance was as unpromising as before. Resorted again to showering, continued it with short intermissions until 2 o'clock, P. M. At this time much better, occasionally asked some questions, wanted drink, &c. Discontinued the water, used other means as before. 3½ o'clock again failing. Showered her as before. 5 o'clock, better. Narcotism seemed fairly passing off. Her bowels were now moved freely, stools contained but little blood. Continued showering at intervals until 4 o'clock next morning. Soporific symptoms had now entirely disappeared. Head cool, tongue whitish, and covered with viscid mucus, rather flighty, asks irrelevant questions, respiration good, pulse however weak and wavy, abdomen somewhat tender, but the grave dysenteric symptoms had disappeared. Ordered small doses of *Hyd cum creta*, with Dover powders, infusion of valerian and ammonia. To be carefully watched. During the next two days there was great nervous debility, with occasional transient paroxysms of reactive fever. The dysenteric symptoms did not reappear, and she soon completely recovered.

The points of particular interest to me in this case are, 1st, The failure of every curative means resorted to except the showering with cold water. Without this efficient aid, her march to death was rapid and inevitable. Congestion of the brain being the dominant toxical effect, seemed to demand that special remedy.

2d. The sudden termination of dysentery. This it is evident could not have occurred had the disease been of long continuance. Structural injuries in the latter case would have prevented it. But it may be asked, would extreme narcotism be equally successful in every recent case of this disease? Several of the other children of the same family were attacked soon afterwards, all of whom passed through a tedious course, and a precarious convalescence. This question, I presume, none will be disposed to answer by testing the utility of the experiment.

P. S. About the time of the occurrence of the above case, or perhaps a little before, I was informed by J. O. Skinner, M. D., of Charleston, that a child in that town two years of age, lost its life by taking eight drops of Tinc. Opii. with Castor oil, given by its mother on going to bed. No medical aid was resorted to.

